# When Sodium Falls With Pineal Gland Involvement: Severe Syndrome of Inappropriate Antidiuretic Hormone Secretion (SIADH) as a Presenting Manifestation of CD5-Positive Primary CNS Large B-cell Lymphoma

**DOI:** 10.7759/cureus.106854

**Published:** 2026-04-11

**Authors:** Daniela Prado Escobar, Kian Memari, Ana Villaverde, Shane Williams, Peter Cohen, Lissette P Lazo

**Affiliations:** 1 Internal Medicine, Nova Southeastern University Dr. Kiran C. Patel College of Osteopathic Medicine, Clearwater, USA; 2 Family Medicine, Palmetto General Hospital, Hialeah, USA; 3 Internal Medicine, Palmetto General Hospital, Hialeah, USA; 4 Family Medicine, Nova Southeastern University Dr. Kiran C. Patel College of Osteopathic Medicine, Fort Lauderdale, USA

**Keywords:** brain biopsy, cd5-positive diffuse large b-cell lymphoma, central nervous system lymphoma, hyponatremia, pineal region mass, primary central nervous system lymphoma, siadh

## Abstract

Primary CNS lymphoma (PCNSL) is an uncommon extranodal non-Hodgkin lymphoma that often presents with nonspecific neurologic symptoms and may be complicated by metabolic derangements. Hyponatremia due to syndrome of inappropriate antidiuretic hormone secretion (SIADH) is a clinically significant but underrecognized manifestation of CNS pathology, and presentation as severe symptomatic hyponatremia in pineal-region PCNSL is particularly unusual. We report the case of a 76-year-old man with a biopsy-proven pineal-region mass who presented with severe symptomatic hyponatremia and progressive encephalopathy. Histopathologic evaluation demonstrated a CD5-positive large B-cell lymphoma with a markedly elevated proliferative index and no evidence of double-hit or triple-hit lymphoma on fluorescence in situ hybridization. The biochemical profile demonstrated hypotonic hyponatremia with inappropriately concentrated urine and elevated urine sodium, supporting SIADH in the setting of intracranial malignancy. The patient required intensive care unit monitoring and was treated with fluid restriction, sodium supplementation, oral urea, and carefully monitored hypertonic saline, resulting in gradual correction of serum sodium and clinical stabilization. This case illustrates how severe SIADH may serve as the presenting clinical manifestation of deep midline PCNSL and underscores the importance of integrating neuroimaging, neuropathology, and electrolyte physiology in medically complex neuro-oncologic presentations.

## Introduction

Primary CNS lymphoma (PCNSL) is a rare extranodal large B-cell lymphoma confined to the brain, spinal cord, leptomeninges, or vitreoretinal compartment in the absence of systemic disease at diagnosis. It accounts for approximately 4% of primary brain tumors and 4-6% of extranodal lymphomas, with incidence increasing in older adults [1,2]. Most cases are histologically diffuse large B-cell lymphomas and frequently involve deep periventricular or midline structures, producing neurocognitive decline, gait disturbance, focal neurologic deficits, or symptoms related to obstructive hydrocephalus and elevated intracranial pressure [1,2].

Pineal-region involvement is distinctly uncommon and broadens the differential diagnosis to include germ cell tumors, gliomas, metastatic lesions, and other pineal-region neoplasms [3]. Tissue diagnosis is therefore essential. Immunophenotypically, PCNSL typically expresses pan-B-cell markers such as CD20 and PAX5, along with activation-associated markers including MUM1, and demonstrates high proliferative indices [1,2]. CD5-positive PCNSL represents a rare subset that may carry distinct biologic behavior and has been associated with more aggressive large B-cell lymphoma biology in other settings, although its prognostic implications in CNS-limited disease remain under investigation [4,5].

Hyponatremia is a common electrolyte disturbance in oncologic patients, and syndrome of inappropriate antidiuretic hormone secretion (SIADH) is the most frequent cause of euvolemic hypotonic hyponatremia [6,7]. CNS pathology, including intracranial malignancy and neurosurgical manipulation, is a well-established trigger for SIADH [6,7]. In PCNSL, SIADH has been reported particularly in association with deep midline lesions, suggesting that lesion location may influence neuroendocrine dysregulation [8]. However, severe symptomatic hyponatremia as the dominant presenting manifestation of a pineal-region CD5-positive PCNSL remains unusual and clinically important because metabolic abnormalities may obscure the underlying oncologic diagnosis.

We report a case of biopsy-proven CD5-positive high-grade large B-cell lymphoma of the pineal region presenting with profound SIADH-related hyponatremia requiring intensive care management [9].

## Case presentation

A 76-year-old man with hypertension, hyperlipidemia, type 2 diabetes mellitus, and a recent stereotactic endoscopic intraventricular biopsy of a pineal-region brain mass was referred to the ED after outpatient laboratory testing demonstrated severe hyponatremia. Family members reported fluctuating mentation, lethargy, dizziness, generalized weakness, and progressive functional decline over the several days preceding admission. On presentation, he was awake but oriented only to person, approximating his recent neurologic baseline but with clear worsening confusion. Neurologic examination demonstrated generalized bilateral upper- and lower-extremity weakness without new focal cranial nerve deficits. The severity of his altered mentation was considered disproportionate to the known stable mass alone and was clinically linked to the serum sodium concentration of 118 mEq/L, raising concern for acute metabolic worsening superimposed on underlying intracranial disease.

Initial laboratory evaluation revealed a serum sodium concentration of 118 mEq/L, serum osmolality of 266 mOsm/kg, urine sodium of 62 mEq/L, and urine osmolality of 514 mOsm/kg, establishing hypotonic hyponatremia with inappropriately concentrated urine (Table 1). Serum creatinine was normal, glucose was only mildly elevated and insufficient to account for the degree of hyponatremia, morning cortisol was within normal limits, and thyroid-stimulating hormone with free thyroxine was normal, excluding adrenal insufficiency and hypothyroidism as competing etiologies. Medication review did not reveal a plausible offending drug. In the setting of euvolemic examination, recent CNS biopsy, and a known deep midline intracranial malignancy, the overall biochemical and clinical profile supported SIADH.

**Table 1:** Laboratory investigations supporting SIADH Laboratory findings demonstrate hypotonic hyponatremia with inappropriately concentrated urine and elevated urine sodium, supporting SIADH in the appropriate clinical context. SIADH: syndrome of inappropriate antidiuretic hormone secretion

Parameter	Patient value	Reference range	Interpretation
Sodium (mEq/L)	118	135-145	Severely decreased
Serum osmolality (mOsm/kg)	266	275-295	Decreased
Urine sodium (mEq/L)	62	Variable	Inappropriately elevated for hypotonic hyponatremia
Urine osmolality (mOsm/kg)	514	50-1200	Inappropriately concentrated
Creatinine (mg/dL)	1	0.6-1.3	Within normal limits
Glucose (mg/dL)	112	70-100	Mildly elevated
Morning cortisol (µg/dL)	16.8	5-25	Within normal limits
Thyroid-stimulating hormone (µIU/mL)	2.1	0.4-4.5	Within normal limits
Free thyroxine (ng/dL)	1.1	0.8-1.8	Within normal limits

**Figure 1:**
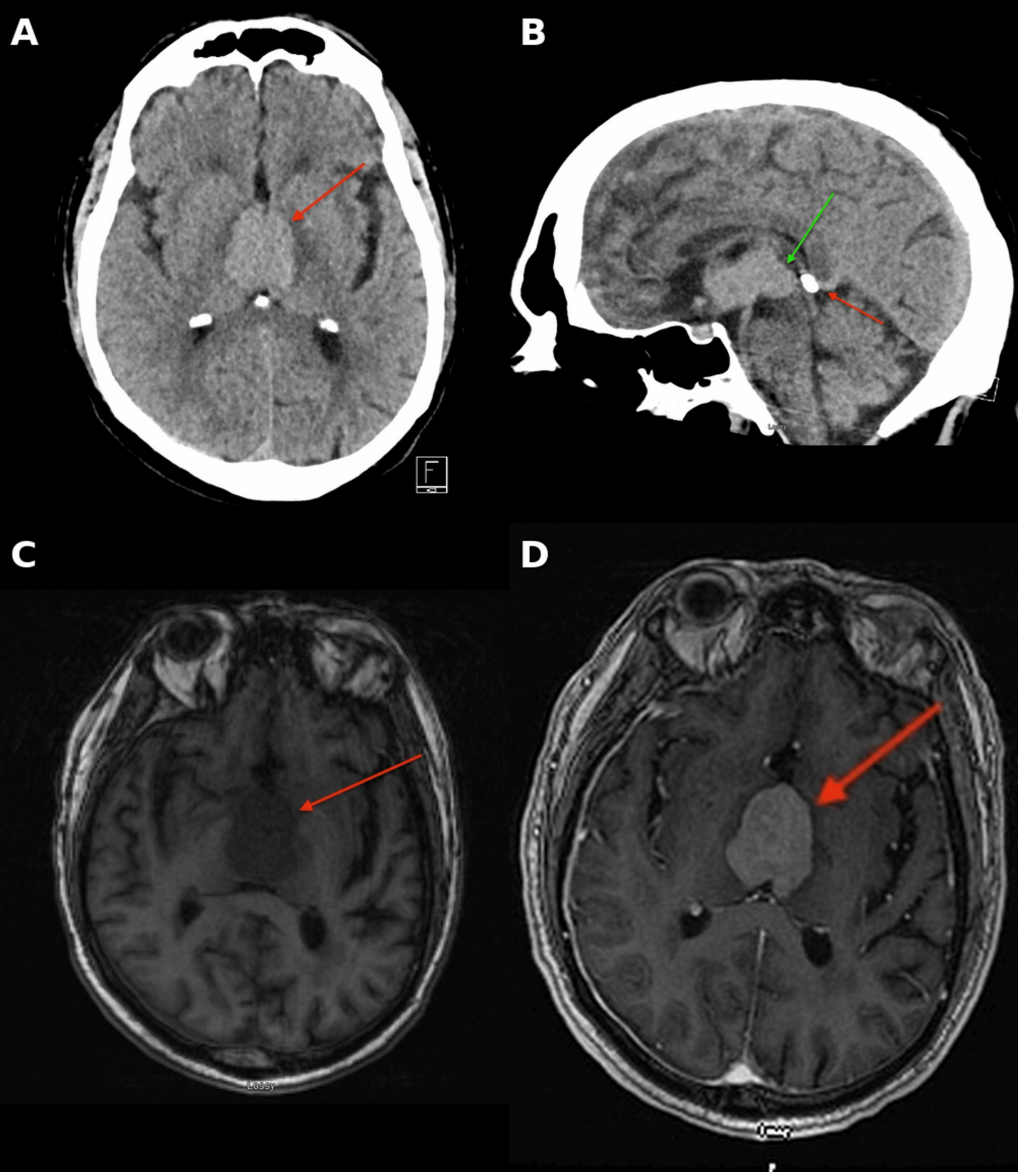
Multimodal neuroimaging demonstrating pineal-region lymphoma (A) Axial noncontrast CT demonstrating a pineal-region mass (red arrow) anterior to the calcified pineal gland with partial effacement of the posterior third ventricle. (B) Sagittal noncontrast CT showing the soft-tissue density mass (green arrow) anterior to the calcified pineal gland (red arrow), delineating its craniocaudal extent. (C) Axial T1-weighted MRI without contrast demonstrating the midline pineal-region lesion (red arrow), biopsy-proven high-grade B-cell lymphoma. (D) Axial fluid-attenuated inversion recovery MRI post-contrast confirming the centrally located tumor (red arrow) within the posterior third ventricle. The deep midline location adjacent to the posterior third ventricle is clinically relevant because local mass effect and associated neuroendocrine pathway disruption may contribute to inappropriate antidiuretic hormone secretion.

**Figure 2:**
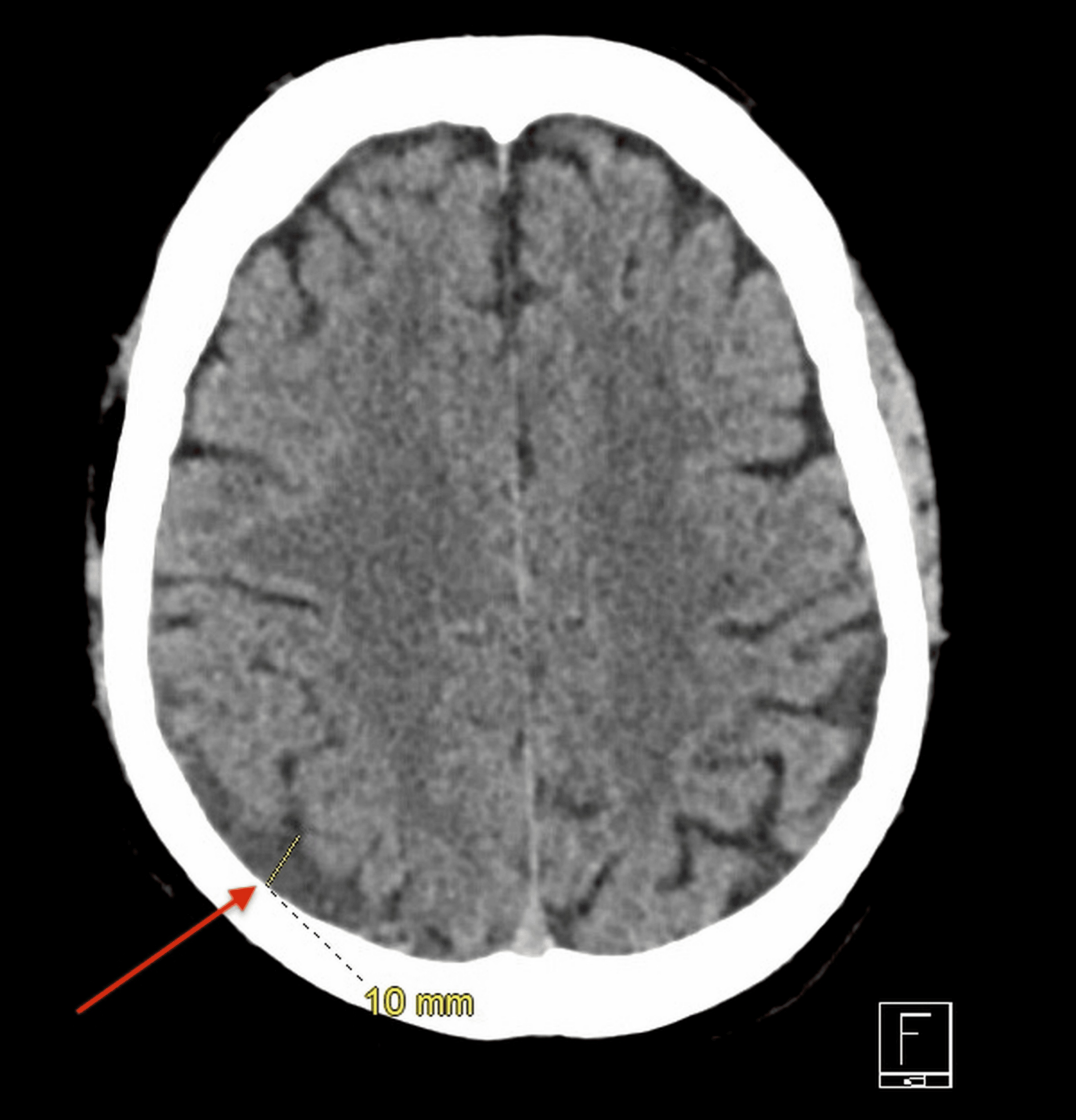
Noncontrast CT of the brain demonstrating a chronic subdural hematoma Axial CT demonstrating a small chronic right posterior parietal subdural hematoma (red arrow) measuring approximately 10 mm in maximal thickness, with internal punctate hyperdensities representing calcification versus a small acute-on-chronic hemorrhagic component. This finding was radiographically distinct from the pineal-region lesion and was not considered the primary driver of the patient’s endocrine presentation.

Noncontrast head CT and MRI demonstrated a pineal-region mass located anterior to the calcified pineal gland, compatible with the known biopsy-proven lesion (Figure 1). A small right posterior parietal chronic subdural hematoma measuring approximately 10 mm was also identified (Figure 2). Subsequent brain MRI, reviewed by the neurology team, demonstrated stability without new acute intracranial abnormalities.

The diagnostic cornerstone of this case was the stereotactic brain tumor biopsy. Histopathologic evaluation demonstrated a malignant neoplasm composed of hyperchromatic large blue cells with focally prominent nucleoli and extensive crush artifact, morphologically favoring a high-grade non-Hodgkin lymphoma with large-cell features. Immunohistochemical studies showed the neoplastic cells to be positive for CD20, PAX5, CD5, MUM1, c-Myc, BCL2, and BCL6 and negative for CD10, cyclin D1, CD30, CD3, CD23, and pankeratin. Ki-67 was markedly increased, highlighting up to 90% of neoplastic nuclei. Additional studies demonstrated negativity for SOX11 and Epstein-Barr virus in situ hybridization.

Fluorescence in situ hybridization showed gains of BCL2 and MYC together with partial deletion/rearrangement of BCL6, but no evidence of MYC or BCL2 rearrangement, and therefore no double-hit or triple-hit lymphoma. The final pathology interpretation was CD5-positive large B-cell lymphoma. The pathology report noted that, if staging excluded systemic lymphoma, the findings would be most consistent with a primary large B-cell lymphoma of immune-privileged sites involving the CNS; alternatively, systemic CD5-positive diffuse large B-cell lymphoma with CNS involvement would remain in the differential, and complete clinical-radiographic staging was recommended.

During admission, hematology-oncology, neurology, neurosurgery, endocrinology, and radiation oncology were consulted (Table 2). Because the immediate priority was correction of severe hyponatremia and neurologic stabilization, no inpatient chemotherapy was initiated. After sodium correction and clinical stabilization, the patient was cleared for discharge with plans for outpatient radiation treatment under neurosurgical and radiation oncology guidance, followed by medical oncology evaluation for high-dose methotrexate-based systemic therapy planning.

**Table 2:** Clinical timeline and management summary SIADH: syndrome of inappropriate antidiuretic hormone secretion

Timepoint	Clinical event
Prior to admission	Progressive cognitive fluctuation, lethargy, dizziness, and weakness in the setting of a known pineal-region mass
Biopsy date	Stereotactic endoscopic intraventricular biopsy performed; pathology demonstrated CD5-positive high-grade large B-cell lymphoma
Outpatient follow-up	Neurosurgeon identified worsening hyponatremia on laboratory testing and referred the patient to the ED
Hospital admission	Serum sodium 118 mEq/L with progressive encephalopathy; admitted to the intensive care unit for severe hypotonic hyponatremia
Inpatient evaluation	CT head confirmed known pineal-region lesion and small chronic right posterior parietal subdural hematoma; endocrine evaluation supported SIADH with urine studies and exclusion of thyroid/adrenal causes
Inpatient management	Fluid restriction, sodium chloride tablets, oral urea, hypertonic saline, serial neurologic examinations, and sodium checks every four hours
Sodium response	Sodium increased from 118 to 124 mEq/L in the first 24 hours, to 129 mEq/L by approximately 48 hours, and to 133 mEq/L by discharge
Pathology review	Immunophenotype: CD20+, PAX5+, CD5+, MUM1+, c-Myc+, BCL2+, BCL6+; Ki-67 up to 90%; Epstein-Barr virus and SOX11 negative; no double/triple hit on fluorescence in situ hybridization
Disposition	Discharged after sodium improved to a safe range, with plans for outpatient radiation and hematology-oncology follow-up

## Discussion

This case illustrates a clinically distinctive presentation of biopsy-proven CD5-positive high-grade large B-cell lymphoma of the pineal region complicated by profound hyponatremia due to SIADH [9]. While PCNSL is uncommon, it should be strongly considered when a deep midline enhancing lesion is accompanied by rapidly evolving neurocognitive decline, especially in older adults [1,2]. What makes this case particularly unusual is that severe symptomatic hyponatremia, rather than focal mass-effect symptoms alone, was the dominant clinical feature prompting intensive care-level management. In that sense, the metabolic complication was not simply a concurrent abnormality but a major presenting manifestation of the pineal-region lymphoma.

Pineal-region involvement is distinctly rare and broadens the radiologic differential, which may delay definitive diagnosis if tissue is not obtained [3]. The neuropathology in this case was particularly informative. The tumor showed a B-cell phenotype with CD20 and PAX5 expression, activation-associated markers including MUM1, a markedly elevated proliferation index, and double-expressor biology with c-Myc and BCL2 protein expression. CD5 positivity is seen only in a minority of diffuse large B-cell lymphomas and has historically been associated with more aggressive systemic disease and frequent CNS involvement [4,5]. More recent pathologic studies suggest that CD5-positive PCNSL may represent a biologically distinct subset within immune-privileged site lymphomas, with possible implications for aggressiveness and treatment responsiveness, although the prognostic impact within true CNS-limited disease remains incompletely defined [4].

From a metabolic standpoint, the sodium disturbance was highly consistent with SIADH. The diagnosis was supported by hypotonic hyponatremia, inappropriately concentrated urine, elevated urine sodium, clinically euvolemic status, and exclusion of adrenal insufficiency, hypothyroidism, clinically significant hyperglycemia, renal failure, and medication-related hyponatremia [6,7]. This more explicit diagnostic framework strengthens the conclusion that SIADH, rather than another cause of hyponatremia, was the principal mechanism. CNS neoplasms may provoke SIADH through disruption of hypothalamic-pituitary signaling, mass effect on deep brain structures, increased intracranial pressure, or perioperative neuroendocrine perturbation following biopsy or resection [7,8]. The coexistence of neurologic decline and electrolyte imbalance creates a diagnostic challenge, as both processes may independently contribute to altered mental status. In this case, severe hyponatremia almost certainly amplified the patient’s lethargy, attention impairment, and functional decline, thereby heightening the urgency of correction.

The pineal location of this lesion is clinically relevant. Although the hypothalamus is classically implicated in SIADH physiology, deep midline tumors adjacent to the third ventricular and diencephalic neuroendocrine axis may perturb antidiuretic hormone regulation indirectly through local mass effect, edema, or altered intracranial signaling [8]. The imaging findings in this case, therefore, have mechanistic importance, not merely descriptive value. Likewise, observational data from adult intracranial tumor populations demonstrate that hyponatremia is more common after neurosurgical intervention and is associated with increased morbidity, prolonged hospitalization, and higher costs of care [10]. These observations support careful electrolyte surveillance in patients with midline CNS tumors, especially after biopsy or when mentation fluctuates.

Management appropriately prioritized neurologic stabilization and controlled sodium correction. Current SIADH literature supports fluid restriction as first-line therapy for chronic or subacute euvolemic hypotonic hyponatremia, with added solute administration such as sodium chloride or urea when restriction alone is insufficient [6,7]. Hypertonic saline is reserved for more severe or symptomatic cases, and correction must be closely monitored to avoid osmotic demyelination syndrome [6,7]. In this patient, correction was intentionally limited to a conservative rate, with sodium checks every four hours and a first-day increase of 6 mEq/L, remaining within accepted safety targets. Definitive oncologic management of PCNSL generally centers on high-dose methotrexate-based therapy with or without rituximab and selected consolidation approaches, while radiotherapy may play a role in older or more medically fragile patients and in bridge-to-systemic-treatment scenarios [1,2]. Because this patient presented primarily with severe hyponatremia and baseline neurologic vulnerability, deferring inpatient chemotherapy until metabolic stabilization was clinically appropriate.

Clinically, this case reinforces the need for vigilance when profound hyponatremia accompanies deep intracranial malignancy. In elderly patients with fluctuating cognition, sodium derangements may be misattributed to chronic neurologic decline or the mass lesion alone. Prompt recognition of SIADH, urgent differentiation from alternative causes of hyponatremia, and timely review of biopsy pathology can substantially alter short-term management and disposition planning [6,7]. This case also illustrates the need to distinguish true PCNSL from systemic CD5-positive diffuse large B-cell lymphoma with CNS involvement, as the therapeutic implications differ.

As a single case, this report should be viewed as illustrative rather than broadly generalizable. The mechanistic link between pineal-region disease and SIADH is strongly supported by the clinical, radiographic, and biochemical findings, although direct physiologic confirmation of hypothalamic involvement is not possible in routine clinical care. Even so, the case provides a clinically meaningful example of how deep midline lymphoma may present primarily through metabolic decompensation.

## Conclusions

This case illustrates that profound hyponatremia due to SIADH may be a clinically significant presenting manifestation of deep midline CD5-positive primary CNS large B-cell lymphoma. In patients with pineal-region or other midline intracranial lesions, severe hyponatremia should prompt careful evaluation for SIADH and close neurologic monitoring. Although this is a single case, it offers a practical reminder that early recognition, controlled sodium correction, and multidisciplinary coordination may improve short-term stabilization and facilitate timely lymphoma-directed treatment.
